# Contribution of GABAergic interneurons to amyloid-β plaque pathology in an APP knock-in mouse model

**DOI:** 10.1186/s13024-019-0356-y

**Published:** 2020-01-08

**Authors:** Heather C. Rice, Gabriele Marcassa, Iordana Chrysidou, Katrien Horré, Tracy L. Young-Pearse, Ulrike C. Müller, Takashi Saito, Takaomi C. Saido, Robert Vassar, Joris de Wit, Bart De Strooper

**Affiliations:** 1 VIB Center for Brain & Disease Research, Leuven, Belgium; 2 Department of Neurosciences, Leuven Brain Institute, KU Leuven, Leuven, Belgium; 3 Present Address: Department of Biochemistry and Molecular Biology, Oklahoma Center for Geroscience, University of Oklahoma Health Sciences Center, Oklahoma City, OK USA; 4 Department of Neurology, Ann Romney Center for Neurologic Diseases, Brigham and Women’s Hospital and Harvard Medical School, Boston, MA USA; 5 Department of Functional Genomics, Institute of Pharmacy and Molecular Biotechnology, Heidelberg University, Heidelberg, Germany; 6 Laboratory for Proteolytic Neuroscience, RIKEN Center for Brain Science, Saitama, Japan; 7 Department of Neurocognitive Science, Nagoya City University Graduate School of Medical Science, Nagoya, Japan; 8 Department of Neurology, Feinberg School of Medicine, Northwestern University, Chicago, USA; 9 UK-Dementia Research Institute at University College London, London, UK

**Keywords:** Alzheimer’s disease, Amyloid precursor protein, Amyloid plaques, APP knock-in, GABAergic neurons, Interneurons, GABA receptor, Hippocampus, BACE1

## Abstract

The amyloid-β (Aβ) peptide, the primary constituent of amyloid plaques found in Alzheimer’s disease (AD) brains, is derived from sequential proteolytic processing of the Amyloid Precursor Protein (APP). However, the contribution of different cell types to Aβ deposition has not yet been examined in an in vivo, non-overexpression system. Here, we show that endogenous APP is highly expressed in a heterogeneous subset of GABAergic interneurons throughout various laminae of the hippocampus, suggesting that these cells may have a profound contribution to AD plaque pathology. We then characterized the laminar distribution of amyloid burden in the hippocampus of an APP knock-in mouse model of AD. To examine the contribution of GABAergic interneurons to plaque pathology, we blocked Aβ production specifically in these cells using a cell type-specific knock-out of BACE1. We found that during early stages of plaque deposition, interneurons contribute to approximately 30% of the total plaque load in the hippocampus. The greatest contribution to plaque load (75%) occurs in the stratum pyramidale of CA1, where plaques in human AD cases are most prevalent and where pyramidal cell bodies and synaptic boutons from perisomatic-targeting interneurons are located. These findings reveal a crucial role of GABAergic interneurons in the pathology of AD. Our study also highlights the necessity of using APP knock-in models to correctly evaluate the cellular contribution to amyloid burden since APP overexpressing transgenic models drive expression in cell types according to the promoter and integration site and not according to physiologically relevant expression mechanisms.

## Background

The biochemical phase of Alzheimer’s disease (AD) is characterized in part by the accumulation and aggregation of the neurotoxic amyloid-β (Aβ) peptide [27], which is generated by sequential proteolytic processing of the Amyloid Precursor Protein (APP). This leads to the complex cellular phase of AD, which involves feedback and feedforward responses of multiple cell types [27]. Distinguishing the contribution of specific cell types to Aβ deposition may provide key insights into the interrelationships between the long-studied biochemical phase and the cellular phase of AD. Excitatory neurons, which generate Aβ in an activity-dependent manner [4, 12], have long been considered the primary source of Aβ deposition in the brain. However, subtypes of fast-spiking GABAergic interneurons are highly active compared to excitatory neurons [7, 13], Moreover, a recent study indicates that cells other than excitatory neurons are a major source of Aβ deposition in a transgenic model of AD [29]. This study was however limited by the fact that APP expression was driven by an artificial prion promoter. Contrary to the long-held perception that APP is a ubiquitously expressed protein, regional and cell-type specific differences of endogenous APP expression, including a striking expression pattern in GABAergic interneurons, have been observed in the mouse hippocampus by us and others [28, 31]. Moreover, GABAergic interneurons were found to be overrepresented in subpopulations of cells that secrete high levels of Aβ in a study that measured Aβ secretion with single-cell resolution from cultured human induced pluripotent stem cell-derived neurons and glia [16].

To circumvent the influence of APP overproduction and mis-patterning, APP knock-in mouse models in which APP is expressed under its endogenous promoter with a humanized Aβ sequence and familial AD mutations have been generated [23, 24]. These *App*^*NL-G-F*^ knock-in mice provide an ideal model to study the contribution of APP expression in GABAergic interneurons of the hippocampus to Aβ generation in mice.

## Results

### APP is prominently expressed in a subset of hippocampal interneurons

**Fig. 1:**
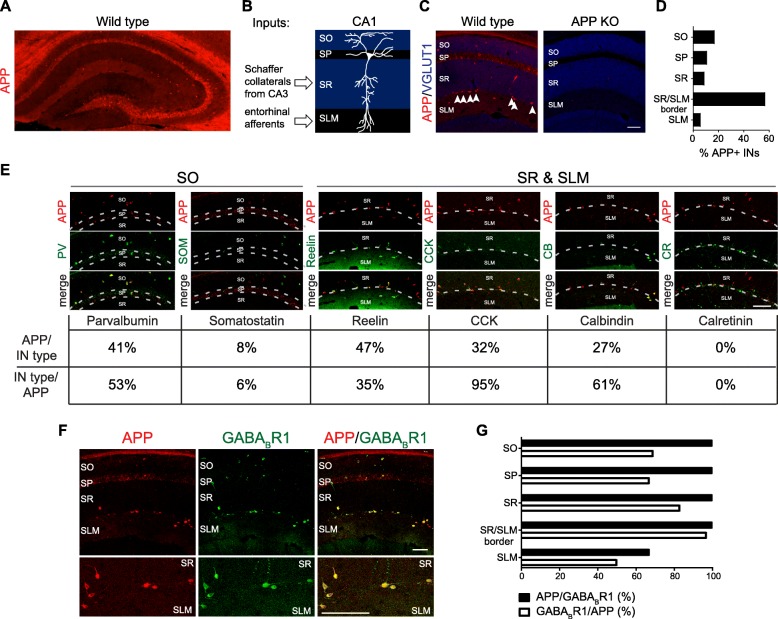
APP expression in interneurons of CA1 hippocampus. **a** Representative confocal image of whole hippocampus from 5-week-old wild type mouse section immunostained for APP. **b** Schematic of the CA1 subfield of the hippocampus. **c** Representative confocal images of hippocampal CA1 subfield of 5-week-old wild type or *App* KO mouse hippocampal sections immunostained for APP and excitatory presynaptic marker VGLUT1. Arrow heads denote APP-positive interneurons at SR/SLM border. **d** Quantification of the laminar distribution of a total of 54 APP-positive interneurons in CA1 examined over 4 sections from 4 different mice. **e** Representative confocal images of 5-week-old wild type mouse hippocampal sections co-stained with APP and interneuron markers (top panels) and quantification of their overlap (bottom panels). For each marker, a total of at least 90 APP-positive interneurons from at least 6 total sections from 2 different mice were examined. **f** Representative confocal images 5-week-old wild type mouse hippocampal sections co-stained with APP and GABA_B_R1. The GABA_B_R1 antibody does not distinguish 1a vs 1b; whereas only 1a is an APP binding partner. **g** Quantification of the overlap between APP-positive and GABA_B_R1-positive GABAergic cells in CA1 laminae. A total of 54 APP-positive cells and 64 GABA_B_R1-positive were examined over 4 sections from 4 different mice. IN = interneuron; SO = stratum oriens; SP = stratum pyramidale; SR = stratum radiatum; SLM = stratum lacunosum-moleculare. Scale bars = 100 μm

*App* mRNA is relatively evenly distributed across *Vgat1*-positive inhibitory neurons and *Vglut1*-positive excitatory neurons in the hippocampus of 5-week old wild type mice (Additional file 1: Figure S1). However, immunohistochemistry of APP (anti- c-terminal APP, B63) in the hippocampus of 5 week-old wild type mice (Fig. 1a) supports a previous report that the distribution of APP protein is prominent in GABAergic interneurons of the hippocampus [14]. There is also strong diffuse staining within the stratum lacunosum-moleculare (SLM) of Cornu Ammonis 1 (CA1). In the CA1 subfield (Fig. 1b), the majority (approximately 60%) of the APP-immunoreactive interneurons resides at the border between the stratum radiatum (SR) (Fig. 1c-d), which receives input from Schaffer collaterals of the CA3 region, and the SLM, which receives input from the entorhinal cortex (Fig. 1b). Approximately 20% of the APP-positive interneurons are located within the stratum oriens (SO) (Fig. 1d), where basal CA1 dendrites reside. We surveyed the neurochemical profile of these APP-positive interneurons (Fig. 1e). Within the SR and SLM, 47% of APP-positive interneurons are Reelin-positive, 32% are Cholecystokinin (CCK)-positive, and 27% are Calbindin-positive (Fig. 1e). Within the SO, 41% of APP-positive interneurons are Parvalbumin-positive (Fig. 1e). However, not all interneurons are immunoreactive for APP. Within the SR and SLM, none of the Calretinin-positive cells are APP-positive, and only 35% of Reelin-positive cells and 61% of Calbindin-positive cells are immunoreactive for APP (Fig. 1e). Within the SO, very few of the Somatostatin-positive cells are APP-positive (8%) and 53% of the Parvalbumin-positive cells are APP-positive (Fig. 1e). CCK-positive cells had the greatest overlap with APP-positive cells, with 95% of CCK-positive cells being also APP-positive across all CA1 laminae (Fig. 1e). Together, we observe that APP has striking expression in a heterogeneous subset of interneurons.

The prominent APP expression in a subset of interneurons suggests that APP function may be important in these cell types. Therefore, we examined the co-expression of APP with γ-aminobutyric acid type B receptor subunit 1(GABA_B_R1) (Fig. 1e), which functionally interacts with the APP ectodomain to regulate presynaptic inhibition [5, 22] and is reported to label a neurochemically heterogeneous subset of interneurons [26]. All APP-positive cells at the SR/SLM border (100%) and in the SO (100%) are GABA_B_R1-positive (Fig. 1f). Conversely, at the SR/SLM border 97% of GABA_B_R1-positive cells are APP-positive, and in the SO 70% of GABA_B_R1-positive cells are APP-positive (Fig. 1f). These findings indicate that the heterogeneous population of APP-positive interneurons strongly but not completely co-expresses its functional binding partner, GABA_B_R1.

### Laminar distribution of amyloid plaques in the hippocampus of an APP knock-in mouse model

**Fig. 2:**
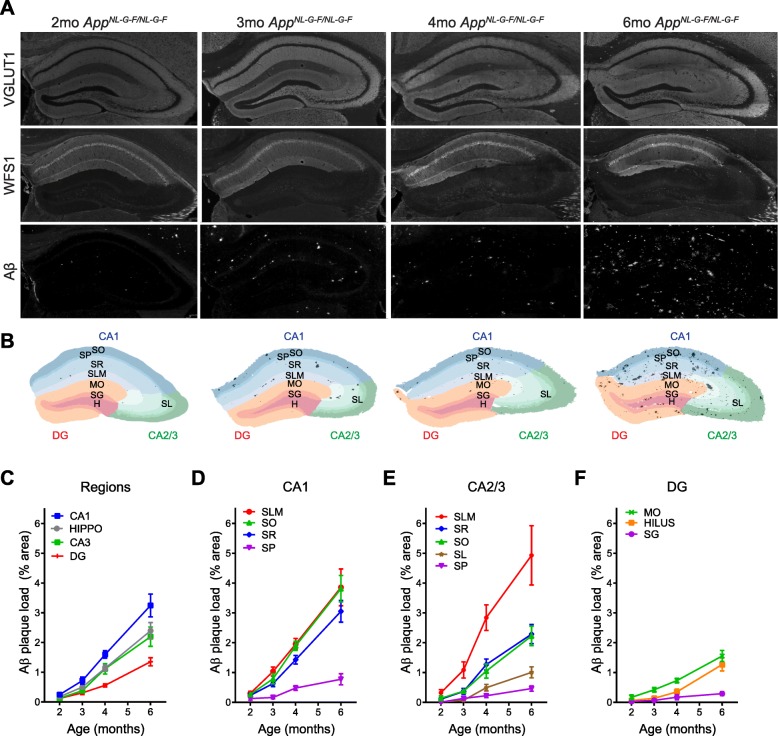
Laminar distribution of Aβ plaques in the hippocampus of an APP knock-in mouse model. **a** Representative images of 2, 3, 4, and 6-month old APP^NL-G-F/NL-G-F^ mouse hippocampal sections immunostained for VGLUT1 (to distinguish laminae), WFS1 (to distinguish subfields), and 6E10 (for Aβ plaques). **b** Corresponding masks used to quantify laminar plaque load. **c-f** Quantification of the Aβ plaque load in hippocampal subfields **c** and laminae of CA1 **d**, CA2/3 **e**, and dentate gyrus (DG) **f**. Graphs show means ± SEM. (*n* = 7 mice). SO = stratum oriens; SP = stratum pyramidale; SR = stratum radiatum; SLM = stratum lacunosum-moleculare; MO = molecular layer; SG = stratum granulosum; H = hilus; DG = dentate gyrus

The striking expression of APP in specific interneuron populations suggests that these interneurons might be major contributors to Aβ pathology in the hippocampus. Therefore, we analyzed plaque distribution in the *App*^*NL-G-F*^ knock-in mouse model [23]. We performed VGLUT1 immunostaining to segment the laminae and WFS1 immunostaining to distinguish CA1 from CA2/3 subfields (Fig. 2a, Additional file 2: Figure S2). Masks for Aβ plaques were created based on Aβ immunostaining (6E10 antibody; Fig. 2a) and combined with the regions of interests for each of the subfields and laminae to quantify Aβ plaque load by percent area (Fig. 2b, Additional file 2: Figure S2).

Using this workflow, we characterized the plaque distribution pattern in 2 month-old (mo), 3mo, 4mo, and 6mo *App* ^*NL-G-F/NL-G-F*^ mice (Fig. 2a). As expected, plaque load in each lamina and subfield increases over time. Plaques begin to appear around 2mo (Fig. 2c-f) and are enriched in the CA1 region of the hippocampus. The dentate gyrus is relatively spared (Fig. 2c, Additional file 3: Figure S3). Plaques are most enriched in the SLM lamina of both CA1 and CA2/3 and in the SO lamina of CA1 (Fig. 2d-e, Additional file 3: Figure S3). In conclusion, we identify some specificity in the distribution of plaques in the hippocampus of *App*^*NL-G-F*^ knock-in model, with the CA1 subfield and the SLM lamina having the highest plaque load.

### Contribution of GABAergic neurons to amyloid pathology in an APP knock-in mouse model

**Fig. 3:**
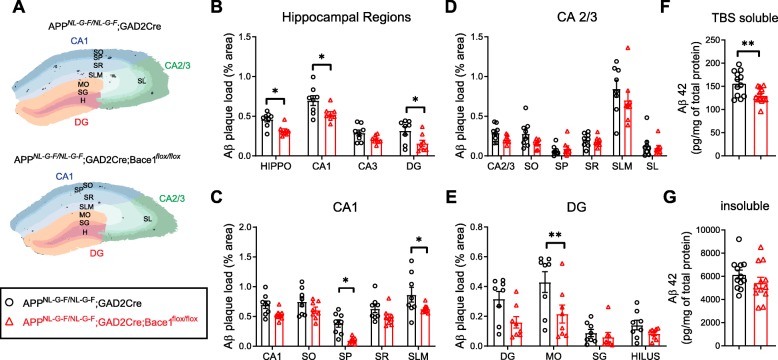
Contribution of GABAergic neurons to Aβ pathology in an APP knock-in mouse model. **a** Representative masks generated by IHC to quantify laminar plaque load in hippocampal sections from *App*^*NL-G-F/NL-G-F*^; *GAD2Cre* (control; black bars) and *App*^*NL-G-F/NL-G-F*^; *GAD2Cre*;*Bace1*^*flox/flox*^ (Bace1cKO in GABAergic neurons; white bars). **b**-**e** Quantification of the Aβ plaque load in hippocampal subfields **b** and laminae of CA1 **c**, CA2/3 **d**, and dentate gyrus (DG) **e** as determined by IHC. Graphs show means ± SEM. (*n* = 8 mice; two-way ANOVA) **f**-**g** Quantification of Aβ42 measured by ELISA from TBS soluble **f** and insoluble **g** fractions of hippocampal brain homogenates. (*n* = 12 mice; student’s t-test) SO = stratum oriens; SP = stratum pyramidale; SR = stratum radiatum; SLM = stratum lacunosum-moleculare; MO = molecular layer; SG = stratum granulosum; H = hilus; DG = dentate gyrus. *P < 0.05, **P < 0.01

To determine the contribution of GABAergic neurons to the initial deposition of Aβ plaques, we blocked Aβ production specifically in GABAergic neurons by conditional knock-out of beta-site amyloid precursor protein cleaving enzyme 1 (BACE1) [18] under the control of Glutamate Decarboxylase 2 (*Gad2-Cre*) in *App*^*NL-G-F/NL-G-F*^ mice. As validation that *Gad2-Cre* targets the appropriate cells with high APP-expression, we observed loss of APP immunoreactivity in GABA_B_R1-positive interneurons in *Aplp2*^*−/−*^ *App* ^*flox/flox*^; *Gad2-Cre* mice compared to *Gad2-Cre* control mice (Additional file 4: Figure S4). Then, we examined plaque load in the hippocampal subfields and laminae of 3mo male *App*^*NL-G-F/NL-G-F*^; *Gad2-Cre*; *Bace1*^*flox/flox*^ mice compared to controls (*App*^*NL-G-F/NL-G-F*^; *Gad2-Cre*). (Fig. 3a-e), which corresponds to an early stage of plaque deposition (Fig. 2). Plaque load in the whole hippocampus is reduced by 31 ± 5% (*n* = 8 mice, *p* < 0.05) (Fig. 3b). The strongest reductions in plaque load are observed in the stratum pyramidale (SP) of CA1 and the molecular layer (MO) of the dentate gyrus. While the reduction in plaque load for the entire CA1 subfield is 24 ± 5% (n = 8 mice, *p* < 0.05) (Fig. 3b), the plaque load in the SP of CA1 falls with 75 ± 5% (*n* = 8 mice, *p* < 0.05) (Fig. 3c). In the latter area, pyramidal cell bodies and synaptic boutons from perisomatic-targeting interneurons are located. In other areas such as the dentate gyrus or the MO where granule cell dendrites ramify, plaque load is reduced by 50 ± 13% (*n* = 8 mice, *p* < 0.05) (Fig. 3b) and 50 ± 14% (*n* = 8 mice, *p* < 0.01) (Fig. 3e), respectively. In addition, we biochemically measured Aβ42 by enzyme-linked immunosorbent assays (ELISA) from the TBS soluble fraction (which represents the soluble non-plaque bound Aβ) (Fig. 3f) and the insoluble fraction (which represents plaque-bound Aβ) (Fig. 3g) of hippocampal homogenates, and a 17 ± 3% (*n* = 12 mice, p < 0.01) reduction of Aβ42 in the soluble fraction was observed. However, during later stages of Aβ deposition, there was not an obvious difference in the plaque load of 6mo *App*^*NL-G-F/NL-G-F*^; *Gad2-Cre*; *Bace1*^*flox/flox*^ mice compared to controls (Additional file 5: Figure S5). Taken together, interneurons contribute to approximately 17% of soluble Aβ and 30% of the total plaque load in the hippocampus and had the greatest effect on plaque load in the SP of CA1 (75%) and the MO of the dentate gyrus (50%) specifically during early stages of plaque deposition.

## Discussion

APP is highly expressed in a subset of GABAergic interneurons in the mouse hippocampus. Parvalbumin, CCK, and Reelin are among the inhibitory neurochemical markers with the greatest overlap with APP-positive cells (Fig. 1e). Interestingly, Reelin interacts with APP [8, 9, 21]. GABA_B_R1, which functionally interacts with the APP ectodomain to regulate presynaptic inhibition [5, 22] has strikingly high overlap with the relatively heterogenous population of APP-positive cells. 98% of APP-positive cells in CA1 are GABA_B_R1-positive (Fig. 1f-g), which is a much greater overlap than any single neurochemical marker for inhibitory neurons examined (Fig. 1e). These expression patterns may hint at the importance of APP function and interactions in populations of GABAergic interneurons. Indeed, GABAergic inhibition and short-term plasticity of GABAergic inputs are impaired with genetic loss of APP [25, 31, 33].

This expression pattern of APP is not expected to be maintained in APP transgenic mouse models but would differ between models depending on the promoter and integration site [10]. Expression of the APP transgene in interneurons has been best studied in the Tg2576 model [11]. While there is prominent APP expression in interneurons of Tg2576 mice, the neurochemical profile of APP-interneurons does not match the profile we found here for endogenous APP. For example, high proportions of somatostatin cells are APP immune-reactive in the Tg2576 mice [11]; whereas we observe almost no overlap of APP with somatostatin cells.

We developed a novel method to analyze plaque load with high spatial resolution of hippocampal laminae and subfields in the *App*^*NL-G-F*^ knock-in mouse model of AD (Additional file 2: Figure S2). Comparing across subfields we found that Aβ plaques are enriched in the CA1 region of the hippocampus compared to the total hippocampus, and the dentate gyrus is relatively spared. This is contrary to APP overexpressing models of AD, in which plaques are enriched in the dentate gyrus [1, 3, 17, 20, 32]. Within the CA1 subfield, we found that the SO and SLM had the highest plaque load in *App*^*NL-G-F*^ mice.

We determined the contribution of GABAergic neurons to amyloid pathology by blocking Aβ production by cell type-specific knock-out of BACE1. This resulted in an overall 30% reduction in total plaque load and 17% reduction in soluble Aβ in the hippocampus. Since GABAergic interneurons are estimated to account for only 10–15% of the total neurons in the hippocampus [19], our findings suggest that GABAergic interneurons, on a per cell basis, contribute at least proportionally to to Aβ production in the hippocampus of *App*^*NL-G-F*^ mice. Possible mechanisms leading to robust secretion of Aβ from interneurons include high-APP expression (Fig. 1) as well as high firing rates of interneurons relative to excitatory neurons [7, 13], since synaptic activity has been shown to promote Aβ generation [4, 12]. Notably, we found that BACE1 knock-out in GABAergic neurons resulted in the greatest reduction (75%) in plaque load in the SP of CA1, where axon terminals of basket-cell interneurons form elaborate basket-like structures on and around the pyramidal cells bodies. These basket-cell interneurons include CCK and Parvalbumin interneurons [19], both of which have high overlap with APP-positive cells. Interestingly, Parvalbumin is known to label highly active ‘fast-spiking’ interneurons, and deficits in Parvalbumin interneurons have been linked to altered network activity in an AD mouse model [30]. Together, our findings reveal a crucial role of GABAergic interneurons in the pathology of AD, particularly in the SP layer of the CA1 hippocampus where plaques in human AD cases are most prevalent [6]. Thus, therapies that modulate activity of GABAergic interneurons could have profound effects on AD pathology.

## Methods

### Animals

All animal experiments were conducted according to the KU Leuven ethical guidelines and approved by the KU Leuven Committee on Animal Care. Generation of the *App*^*NL-G-F*^ mice, *Bace1*^*flox/flox*^, *App* KO, and *Aplp2*^*−/−*^ *App* ^*flox/flox*^ mice were described previously [2, 15, 18, 23]. *GAD2Cre* mice were obtained from Jackson Laboratory (Jax 010802). Both male and female *App* ^*NL-G-F*^ were used in the time course experiments in Fig. 2. Only male mice were used in Fig. 3.

### Histology & Immunohistochemistry

Mice were transcardially perfused with 5 mL of saline solution followed by 10 mL of 4% PFA in PBS. Brains were dissected, post-fixed for 1 h at 4 °C with 4% PFA, and then incubated in 30% sucrose solution. Brains were embedded in OCT (Sakura-Tissue-Tek, 4583) and frozen in isopentane. 16um coronal sections were generated using a cryostat (Nx70, ThermoFisher).

16um coronal sections were post-fixed in 1:1 ice-cold MeOH-Acetone for 10 min, washed with PBS and 0.5% Triton X-100 in PBS (PBS-T), and blocked in PBS-gelatin containing 10% NHS, 1:43 Donkey anti-mouse Fab frabment (Jackson ImmunoResearch, 715–007-003), and 0.5% Triton X-100 for 2 h. Samples were then incubated overnight at 4 °C with primary antibodies in PBS-gelatin containing 5% NHS and 0.5% Triton X-100. Primary antibodies included the following: mouse anti-6E10 (1:1000, BioLegend, 803,003), rabbit anti-WFS1 (1:600, ProteinTech, 11,558–1-AP), guinea pig anti-vGLUT1 (1:5000, Millipore, AB5905), rabbit anti-APP (1:10,000, B63, c-terminal,), mouse anti-Parvalbumin (1:1000, Swant, 235), rat anti-somatostatin (1:500, Millipore, MAB354), mouse anti-Reelin (1:500, CR50, MBL International, D223–3,), mouse anti-CCK (1:250, AbCam, ab37274), mouse anti-Calbindin (1:2500, Swant, 300), guinea pig anti-Calretinin (Calbindin D29k) (1:1000, Synaptic Systems, 214,104), mouse anti-GABABR1 (1:500, NeuroMab, 75–183,). Samples were subsequently washed in 0.5% PBS-T and incubated with secondary antibodies in PBS-gelatin containing 5% NHS and 0.5% Triton X-100, for 2 h at room temperature. Coverslips were mounted using Mowiol mounting medium. Sections were imaged using Leica confocal microscopes (SP5 and SP8) for imaging of interneurons or the Axio Scan.Z1 Slide Scanner (ZEISS) with 20X objective for Aβ plaque analysis.

### Image processing

For quantification of immune-positive cells by IHC: Using ImageJ software, vGLUT1 staining was used to segment hippocampal layers. Manual thresholding was performed and the automatic “Wand (Tracing) Tool” was used to select immunopositive cells and generate masks. Accordingly, APP positive cells are defined as cells with APP immunolabeling above threshold.

For quantification of Aβ plaque load by IHC: Using the ZEN software, single hippocampi were selected and images were exported as TIFF files (8bit, LWZ compression, scalebar). Exported TIFF files were then analyzed using the ImageJ software. Images with vGLUT1 staining were used to segment the hippocampal layers. A manual threshold was applied, and the stratum pyramidale (SP) and stratum granulosum (SG) layers were selected using the automatic “Wand (Tracing) Tool”, while the whole hippocampus was either automatically selected, or manually defined using the “Selection Brush Tool”. Then, the remaining hippocampal laminae were manually segmented. Using the WFS1-staining images, ROIs were refined to separate the Cornu Ammonis (CA) 1 and 2/3 hippocampal subfields. After ROIs were defined, 6E10-staining images were used to create a mask for Aβ plaques. For the Αβ plaque-mask, an automatic threshold was applied (threshold name: “Triangle”), and the particles with size > 10 μm^2 were considered for the mask creation. Finally, ROIs were applied on top of the Aβ plaque-mask and measurements were analyzed per layer for “Area” (area of each layer) and “Area Faction” (fraction covered by plaques).

### Aβ extraction and ELISA

Mice were transcardially perfused with saline, and hippocampi were dissected and flash-frozen. Hippocampi were mechanically homogenized using Fastprep tubes and T-PER Tissue Protein Extraction Reagent (Thermo Fisher Scientific, 78,510) with phosphatase inhibitors (Merck, P0044 and P5726) and cOmplete protease inhibitors (Roche, 11,836,145,001). The TBS soluble fraction was collected as the supernatant after ultracentrifugation (1 h, 4 °C, 55000 rpm; TLA 100.4 rotor, Beckman Coulter). For the insoluble fraction, the pellet was resuspended in 2 volumes (vol: wet weight of tissue) of GuHCl (6 M GuHCl/50 mM Tris-HCl, pH 7.6) with cOmplete protease inhibitors and sonicated for 30s. After 1 h incubation at 25 °C followed by ultracentrifugation (20 min, 70.000 rpm, 4 °C; TLA 100.4 rotor, Beckman Coulter), the supernatant was diluted 12X in GuHCl diluent buffer (20 mM phosphate, 0.4 M NaCl, 2 mM EDTA, 10% Block Ace, 0.2% BSA, 0.0% NaN3, 0.075% CHAPS, pH 7.0) with cOmplete protease inhibitors. Aβ42 levels were quantified on MSD single spot 96 well plates in house coated overnight with JRF Aβ42/26 antibody at 1,5 μg/ml in PBS. Plates were rinsed 5 x with 200 μl/well washing buffer (PBS + 0.05% Tween-20), blocked with 150 μl/well 0.1% casein buffer for 1.5 h at room temperature (600 rpm) and rinsed 5 x with 200 μl/well washing buffer. 25 μl of SULFO-TAG JRF/AbN/25 detection antibody diluted in blocking buffer was mixed with 25 μl of standards (synthetic human Aβ1–42 peptide) or reaction samples diluted in blocking buffer (1/2 dilution for soluble Aβ-fraction and 1/250 for insoluble Aβ-fraction) and loaded 50 μl per well. After overnight incubation at 4 °C, plates were rinsed with washing buffer and 150 μl/well of the 2x MSD Read Buffer T (tris-based buffer containing tripropylamine, purchased from Meso Scale Discovery) was added. Plates were immediately read on MSD Sector Imager 6000.

### RNAscope in situ hybridization

16 μm coronal hippocampal cryosections were obtained from flash-frozen, 5 week old C57BL/6 and *App* KO mouse brains. RNAscope in situ hybridization was performed using the Fluorescent Multiplex Reagent Kit (Advanced Cell Diagnostics, 320,850) following manufacturer’s protocol. Heating steps were performed using the HybEZTM oven (Advanced Cell Diagnostics). Sections were pretreated with Pretreat 4 reagent and hybridized with the following probes: Mm-Slc17a7 (416631), Mm-App-XHs-C2 (519001), Mm-Slc32a1-C3 (319191). After amplification steps, sections were mounted using Prolong Gold Antifade (ThermoScientific). Imaging was performed using a slidescanner microscope (Zeiss Axioscan.Z1) with a 20X air objective. Image processing was performed in ZEN 2.3 lite and FIJI.

## Supplementary information

**Additional file 1: Figure S1.** APP mRNA expression in excitatory and inhibitory neurons of the hippocampus*.* Fluorescent in situ hybridization for *App* (red), the inhibitory neuron marker *Vgat* (green) and the excitatory neuron marker *Vglut1* (blue) mRNAs in wild type and APP KO coronal sections. Insets show CA1 region. Scalebar is 100 μm.

**Additional file 2: Figure S2.** Workflow for quantification of Aβ plaque load across hippocampal laminae. Outline of methods developed to quantify Aβ load across hippocampal laminae by segmenting hippocampal laminae, refining hippocampal subfields, creating masks to define Aβ plaque area, and combing the regions of interest (ROIs) for each subfield and laminae with masks for Aβ plaque area.

**Additional file 3: Figure S3.** Laminar distribution of Aβ plaques in the hippocampus of APP knock-in mice. Graphs detailing the data summarized in Fig. 2c-f. Quantification of the Aβ plaque load in hippocampal subfields and laminae of CA1, CA2/3 and dentate gyrus (DG) of sections from 2, 3, 4, and 6 month old APP^NL-G-F/NL-G-F^ mice. Graphs show means ± SEM. (*n* = 5–7 mice; one-way ANOVA). *P < 0.05, **P < 0.01, **P < 0.001 SO = stratum oriens; SP = stratum pyramidale; SR = stratum radiatum; SLM = stratum lacunosum-moleculare; MO = molecular layer; SG = stratum granulosum; H = hilus; DG = dentate gyrus.

**Additional file 4: Figure S4.** Loss of APP immunoreactivity in GABABR1-positive interneurons in Aplp2^−/−^App ^flox/flox^;Gad2-Cre mice. Representative confocal images of mouse hippocampal sections of Aplp2^−/−^App ^flox/flox^;Gad2-Cre.

**Additional file 5: Figure S5.** Plaque load of 6mo App^NL-G-F/NL-G-F^; Gad2-Cre; Bace1^flox/flox^ mice compared to controls. Quantification of the Aβ plaque load in hippomcapus as determined by IHC. Graphs show means ± SEM. (*n* = 3 mice).

## Data Availability

Raw data is available from the corresponding authors upon reasonable request.

## Abbreviations

AD: Alzheimer’s disease
APP: Amyloid Precursor Protein
Aβ: amyloid-β
BACE1: beta-site amyloid precursor protein cleaving enzyme 1
CA1: Cornu Ammonis 1
CCK: Cholecystokinin
DG: dentate gyrus
ELISA: Enzyme-linked immunosorbent assays
GABA_B_R1: γ-aminobutyric acid type B receptor subunit 1
Gad2: Glutamate Decarboxylase 2
H: Hilus
MO: Molecular layer
mo: Month-old
SG: Stratum granulosum
SLM: Stratum lacunosum-moleculare
SO: Stratum oriens
SP: Stratum pyramidale
SR: Stratum radiatum
