# Supplementary figures and images for: Contribution of GABAergic interneurons to amyloid-β plaque pathology in an APP knock-in mouse model
Source: Mol Neurodegener. 2020 Jan 8;15:3. doi: 10.1186/s13024-019-0356-y (PMC6950898; doi:10.1186/s13024-019-0356-y)

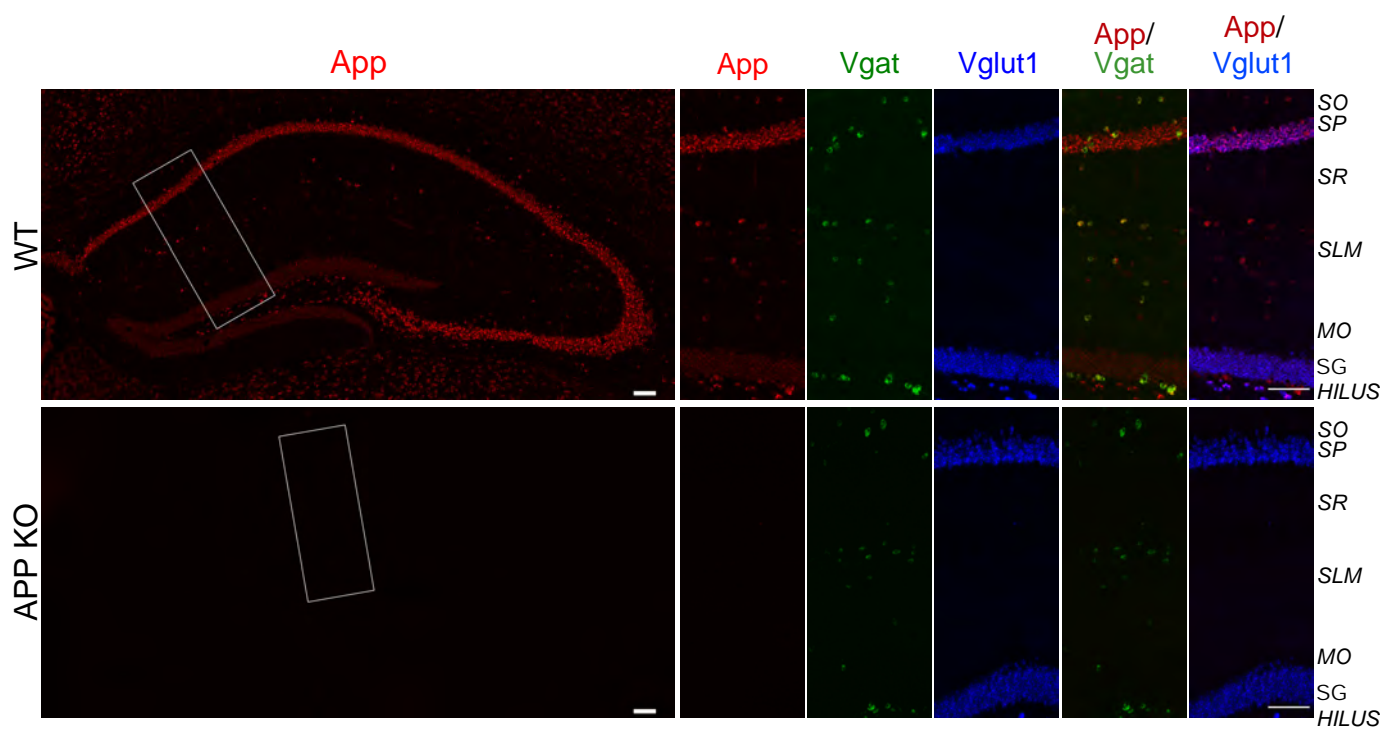

Supplement: Supplementary file 1 — Additional file 1: Figure S1. APP mRNA expression in excitatory and inhibitory neurons of the hippocampus. Fluorescent in situ hybridization for App (red), the inhibitory neuron marker Vgat (green) and the excitatory neuron marker Vglut1 (blue) mRNAs in wild type and APP KO coronal sections. Insets show CA1 region. Scalebar is 100 μm. [file 13024_2019_356_MOESM1_ESM.pdf]

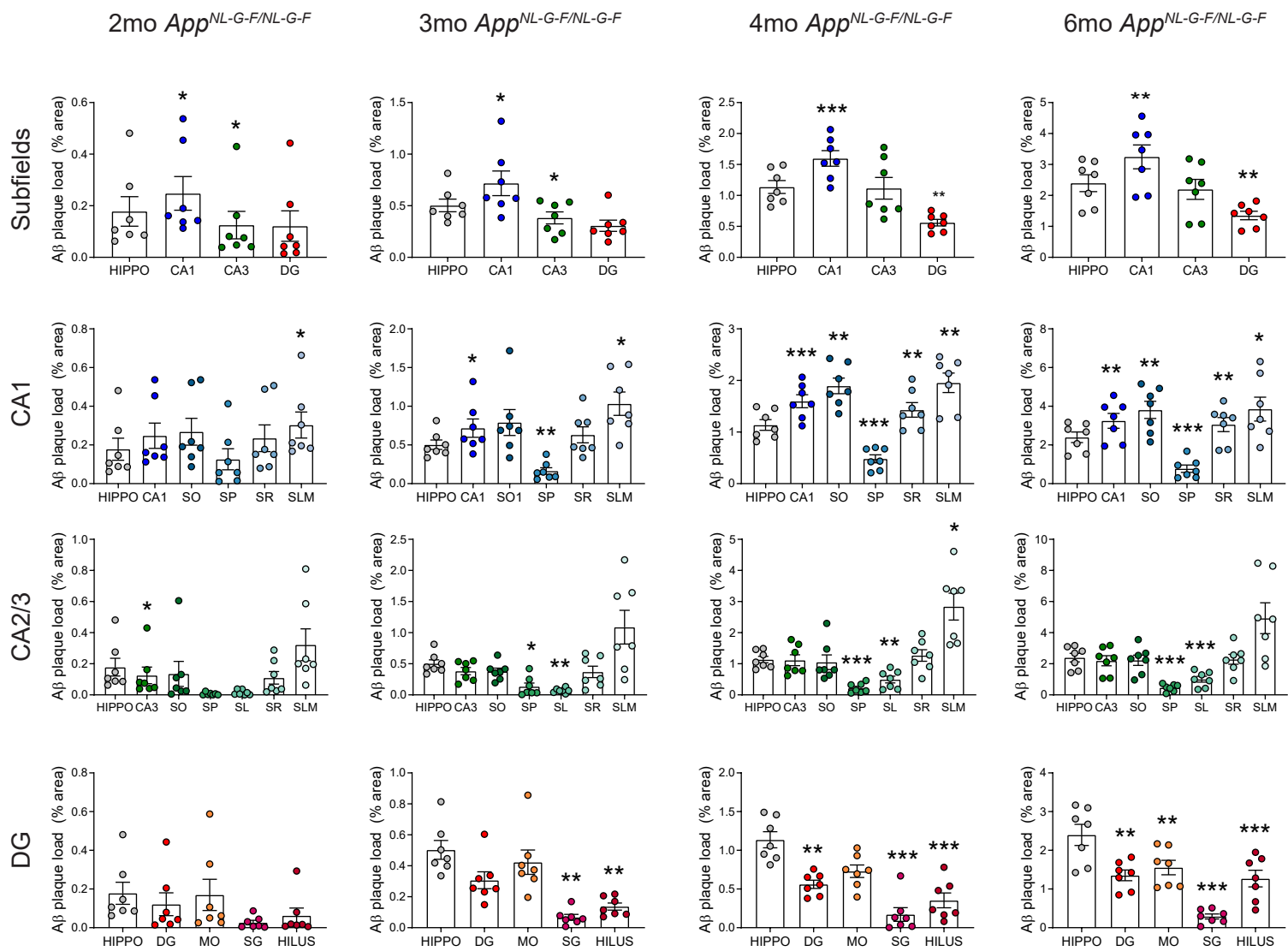

Supplement: Supplementary file 3 — Additional file 3: Figure S3. Laminar distribution of Aβ plaques in the hippocampus of APP knock-in mice. Graphs detailing the data summarized in Fig. 2c-f. Quantification of the Aβ plaque load in hippocampal subfields and laminae of CA1, CA2/3 and dentate gyrus (DG) of sections from 2, 3, 4, and 6 month old APPNL-G-F/NL-G-F mice. Graphs show means ± SEM. (n = 5–7 mice; one-way ANOVA). *P < 0.05, **P < 0.01, **P < 0.001 SO = stratum oriens; SP = stratum pyramidale; SR = stratum radiatum; SLM = stratum lacunosum-moleculare; MO = molecular layer; SG = stratum granulosum; H = hilus; DG = dentate gyrus. [file 13024_2019_356_MOESM3_ESM.pdf]

## GADiCre

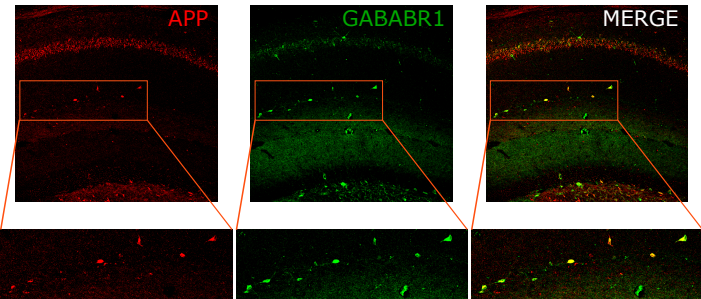

## GADiCre;APP<sup>flox/flox</sup>;APLP2-KO

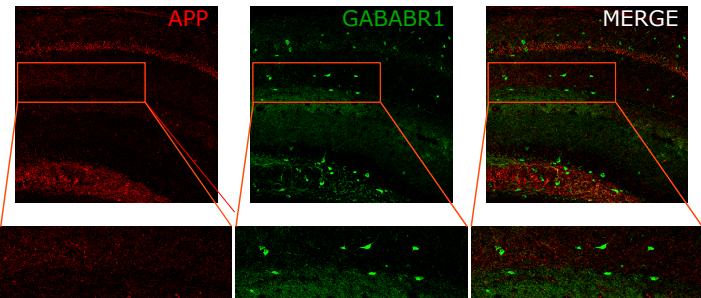

Supplement: Supplementary file 4 — Additional file 4: Figure S4. Loss of APP immunoreactivity in GABABR1-positive interneurons in Aplp2−/−App flox/flox;Gad2-Cre mice. Representative confocal images of mouse hippocampal sections of Aplp2−/−App flox/flox;Gad2-Cre. [file 13024_2019_356_MOESM4_ESM.pdf]
