# Supplementary material for: Contribution of GABAergic interneurons to amyloid-β plaque pathology in an APP knock-in mouse model
Source: Mol Neurodegener. 2020 Jan 8;15:3. doi: 10.1186/s13024-019-0356-y (PMC6950898; doi:10.1186/s13024-019-0356-y)
Supplement: Supplementary file 2 — Additional file 2: Figure S2. Workflow for quantification of Aβ plaque load across hippocampal laminae. Outline of methods developed to quantify Aβ load across hippocampal laminae by segmenting hippocampal laminae, refining hippocampal subfields, creating masks to define Aβ plaque area, and combing the regions of interest (ROIs) for each subfield and laminae with masks for Aβ plaque area. [file 13024_2019_356_MOESM2_ESM.pdf]

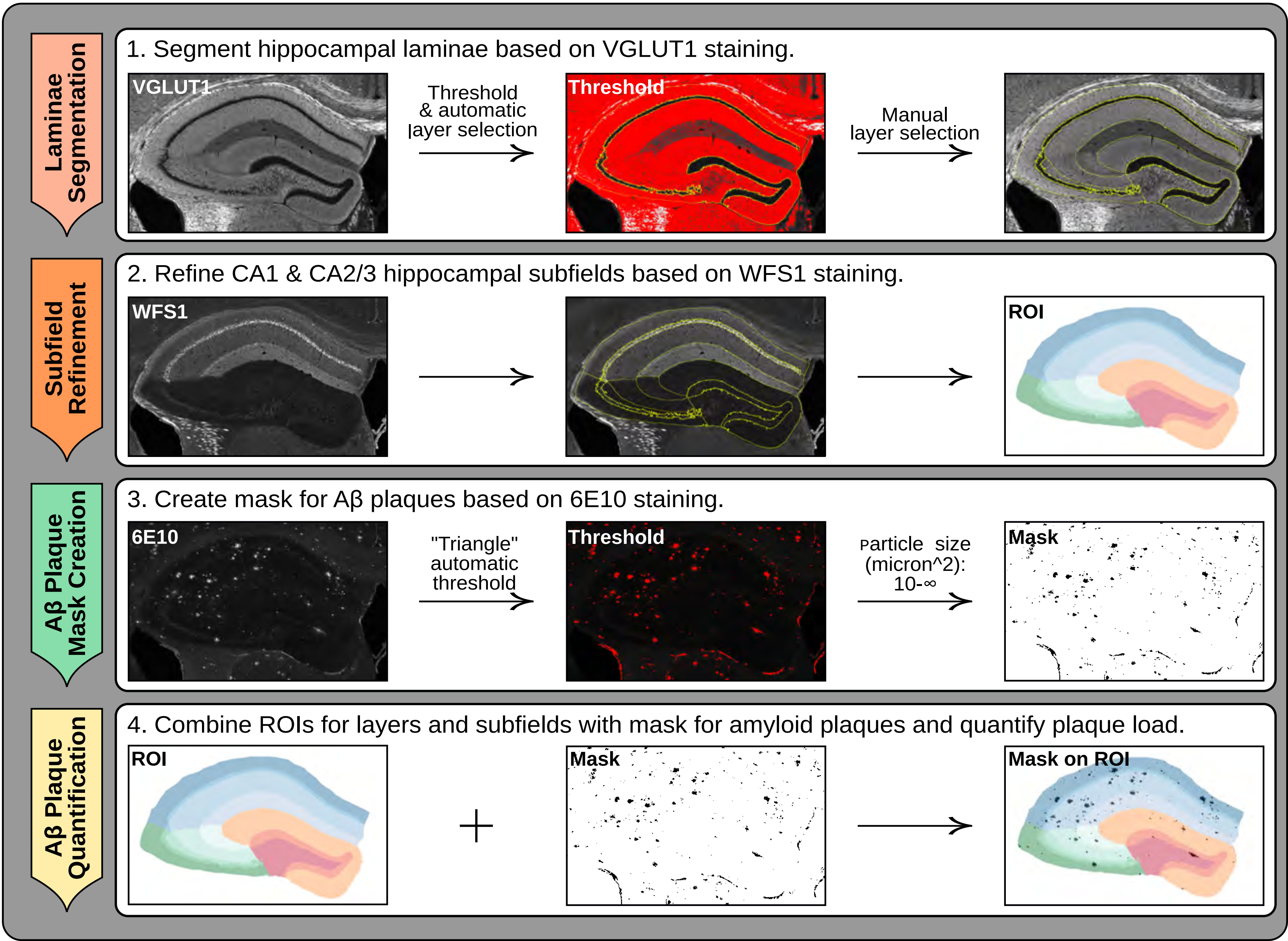
Laminae Segmentation
1. Segment hippocampal laminae based on VGLUT1 staining.
VGLUT1
Threshold & automatic layer selection
Threshold
Manual layer selection
Subfield Refinement
2. Refine CA1 & CA2/3 hippocampal subfields based on WFS1 staining.
WFS1
ROI
Aβ Plaque Mask Creation
3. Create mask for Aβ plaques based on 6E10 staining.
6E10
"Triangle" automatic threshold
Threshold
Particle size (micron^2): 10-∞
Mask
Aβ Plaque Quantification
4. Combine ROIs for layers and subfields with mask for amyloid plaques and quantify plaque load.
ROI
Mask
Mask on ROI
