# Supplementary material for: Contribution of GABAergic interneurons to amyloid-β plaque pathology in an APP knock-in mouse model
Source: Mol Neurodegener. 2020 Jan 8;15:3. doi: 10.1186/s13024-019-0356-y (PMC6950898; doi:10.1186/s13024-019-0356-y)
Supplement: Supplementary file 5 — Additional file 5: Figure S5. Plaque load of 6mo AppNL-G-F/NL-G-F; Gad2-Cre; Bace1flox/flox mice compared to controls. Quantification of the Aβ plaque load in hippomcapus as determined by IHC. Graphs show means ± SEM. (n = 3 mice). [file 13024_2019_356_MOESM5_ESM.pdf]

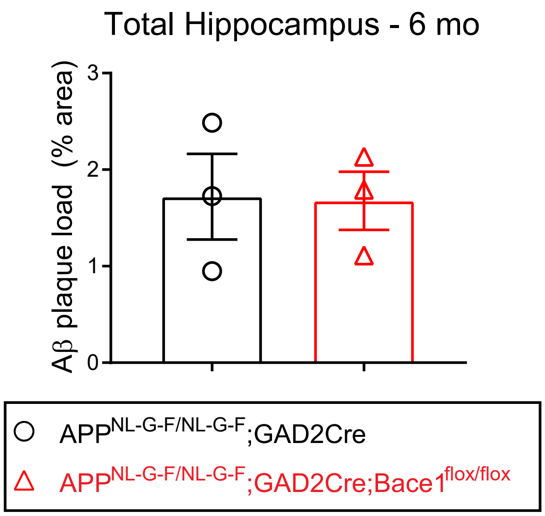
Total Hippocampus - 6 mo
Aβ plaque load (% area)
0
1
2
3
APP^NL-G-F/NL-G-F;GAD2Cre
APP^NL-G-F/NL-G-F;GAD2Cre;Bace1^flox/flox
